# Effect of Ferredoxin Receptor FusA on the Virulence Mechanism of *Pseudomonas plecoglossicida*

**DOI:** 10.3389/fcimb.2022.808800

**Published:** 2022-03-22

**Authors:** Rongchao He, Jiajia Wang, Miaozhen Lin, Jing Tian, Bi Wu, Xiaohan Tan, Jianchuan Zhou, Jiachen Zhang, Qingpi Yan, Lixing Huang

**Affiliations:** ^1^ Fisheries College, Key Laboratory of Healthy Mariculture for the East China Sea, Ministry of Agriculture, Jimei University, Xiamen, China; ^2^ College of Food and Biological Engineering, Jimei University, Xiamen, China

**Keywords:** visceral white spot disease, *Pseudomonas plecoglossicida*, virulence, *fusA*, environment adaptation

## Abstract

*Pseudomonas plecoglossicida* is an aerobic Gram-negative bacterium, which is the pathogen of “Visceral white spot disease” in large yellow croaker. *P. plecoglossicida* is a temperature-dependent bacterial pathogen in fish, which not only reduces the yield of large yellow croaker but also causes continuous transmission of the disease, seriously endangering the healthy development of fisheries. In this study, a mutant strain of *fusA* was constructed using homologous recombination technology. The results showed that knockout of *P. plecoglossicida fusA* significantly affected the ability of growth, adhesion, and biofilm formation. Temperature, pH, H_2_O_2_, heavy metals, and the iron-chelating agent were used to treat the wild type of *P. plecoglossicida*; the results showed that the expression of *fusA* was significantly reduced at 4°C, 12°C, and 37°C. The expression of *fusA* was significantly increased at pH 4 and 5. Cu^2+^ has a significant inducing effect on the expression of *fusA*, but Pb^2+^ has no obvious effect; the expression of *fusA* was significantly upregulated under different concentrations of H_2_O_2_. The expression of the *fusA* gene was significantly upregulated in the 0.5~4-μmol/l iron-chelating agent. The expression level of the *fusA* gene was significantly upregulated after the logarithmic phase. It was suggested that *fusA* included in the TBDR family not only was involved in the transport of ferredoxin but also played important roles in the pathogenicity and environment adaptation of *P. plecoglossicida*.

## 1 Introduction

*Pseudomonas plecoglossicida* was firstly isolated from ayu (*Plecoglossus altivelis*) (Nishimori et al., 2000), which was a gram-negative aerobic rod-shaped bacterium responsible for the bacterial hemorrhagic ascites (Wakabayashi et al., 1996; Kuehn and Kesty, 2005; Dumetz et al., 2007; Zhang et al., 2014). At present, *P. plecoglossicida* has been reported to be associated with diseases in a variety of marine fish such as rainbow trout (*Oncorhynchus mykiss*), large yellow croaker (*Pseudosciaena crocea*), and orange spotted grouper (*Epinephelus coioides*) (Lin et al., 2021). Previous studies have shown that *P. plecoglossicida* is used to degrade industrial waste that pollutes the environment. For example, *P. plecoglossicida* TED35 is used to degrade tobacco waste containing the alkaloid nicotine (Raman et al., 2014) and *P. plecoglossicida* NyZ12 uses cyclohexane (CHAM) as a source of carbon and nitrogen to degrade cyclohexane (Li et al., 2015). Because *P. plecoglossicida* mainly infects fish’s kidney, spleen, and other internal organs, with white nodules on the surface as the main symptom and a very high mortality rate, it is therefore called “Visceral white spot disease” (Tao et al., 2016). Artificial infection with *P. plecoglossicida* caused “Visceral white spot disease” in the internal organs of *P. crocea* and *E. coioides* at 16°C–19°C but not at 7°C–12°C and 24°C–28°C (Sun et al., 2018). With previous transcriptomic analysis, it was also confirmed that *P. plecoglossicida* was a temperature-dependent pathogenic bacteria (Zhang et al., 2018a; Jiao et al., 2021).

The large yellow croaker is mainly distributed in Southeast China. It is one of the most important economically maricultured fish species in China, and its yield has been the first in China for many years (Chen et al., 2008; Liu and de Mitcheson, 2008; Wu et al., 2015). In the process of sea cage culture of large yellow croaker, “Visceral white spot disease” caused by *P. plecoglossicida* is one of the diseases with the highest mortality, causing huge aquatic economic losses to southeast coastal areas such as Fujian and Zhejiang (, Watts et al., 2001; Zhang et al., 2007; Martins et al., 2011; Hu et al., 2014; Lin et al., 2021). Therefore, it is of great significance to study the virulence mechanism of *P. plecoglossicida.*

Iron is an indispensable nutrient element for all living organisms in the world, and competing for scarce nutrients is a “required course” for most microorganisms (Lim, 2010). In Gram-negative bacteria, the outer membrane receptors of the TonB-dependent receptor (TBDR) family perform their functions by binding microorganisms with high affinity to remove iron carriers and iron-containing host proteins such as lactoferrin, transferrin, and hemoglobin (Schaible and Kaufmann, 2004; Fujita et al., 2020). The transport from nucleus to periplasm also depends on TBDRs. TBDRs work with the nucleus through a highly specialized extracellular matrix structure, with an external loop of a 22-stranded transmembrane channel β-barrel. After these initial interactions, the pipeline provides a conduit through the outer membrane for iron or the iron carrier complex (Pollet et al., 2021). TBDRs are known to play an important role in the pathogenesis of host infection (Cornelissen et al., 1998; Noinaj et al., 2012; Ollerton et al., 2014). *fusA* is a newly discovered member of the TonB-dependent receptor family, which is involved in virulence regulation at the transcriptional level of some pathogenic bacteria. *fusA* was found to be used to obtain iron from plant ferridoxins in plant pathogenic *Pectobacterium* spp (Díaz-Sánchez et al., 2012). Violeta Díaz-Sánchez et al. found that the *fusA* gene was related to the pathogenic function of *Fusarium fujikuroi*. Nore´n found that the *fusA* gene was important for bacterial protein synthesis and was associated with drug resistance of *Clostridium difficile* (Noreín et al., 2007). In another study, the resistance of *Corynebacterium glutamicum* and *Brevibacterium flavum* to fusidic acid was related to *fusA* gene mutation, and all clones containing *fusA* gene mutation produced 10% more lysine than their parents (Tokmakova et al., 2017). In addition, through dual RNA-seq (Luo et al., 2019), we found that *fusA* might be a virulence gene that played an important role in the process of *P. plecoglossicida* infection, but the specific mechanism is unclear. Therefore, it is of far-reaching significance to explore the function of *fusA* and its possibility as a target for slowing down “visceral white spot disease” or becoming a live attenuated vaccine.

In order to better understand the function of *fusA* in *P. plecoglossicida*, we constructed the *fusA* knockout strain and investigated the effects of the *fusA* mutant on the ability of growth, adhesion, biofilm formation, and environment adaptation of *P. plecoglossicida*.

## 2 Materials and Methods

### 2.1 Bacterial Strains and Culture Conditions

The pathogenic strain isolated from the spleen of large yellow croaker with visceral white spot disease was later confirmed as *P. plecoglossicida* (NZBD9) (Huang et al., 2018; Zhong et al., 2021). The strain was cultured in LB medium at 220 rpm at 28°C (He et al., 2020). *Escherichia coli* DH5α was obtained from TransGen Biotech (Beijing, China) and cultured in LB medium (37°C, 220 rpm). DH5α pCM 130 and DH5α pKD 46 were preserved in the laboratory.

### 2.2 Sequence Alignment Analysis, Phylogenetic Tree Construction, and Protein Structure Prediction

The amino acid sequence of the *fusA* gene of *P. plecoglossicida* was compared with *Escherichia coli* str. K-12 substr. MG1655 (a), *Staphylococcus argenteus* (strain: MSHR1132) (b), *Vibrio alginolyticus* (strain: FDAARGOS_97) (c), *Pseudomonas lactis* (strain: SS101) (d), *Listeria seeligeri serovar 1/2b* str. SLCC3954 (e), *Pseudomonas plecoglossicida* (strain: NZBD9) (f), *Pseudomonas syringae pv.* tomato str. DC3000 (g), *Vibrio harveyi* (strain: ATCC 33843 (392 [MAV])) (h), *Aeromonas hydrophila* (strain: OnP3.1) (i), and *Pseudomonas aeruginosa* PA96 (j) by ClustalW and ENDscript2.x/ESPript3.x to map the sequence alignment results; the phylogenetic tree was constructed by MEGA7. The protein structure of FusA of *P. plecoglossicida* was predicted by I-TASSER.

### 2.3 Construction of Mutant Strain of *P. plecoglossicida*

The *fusA* knockout strain of *P. plecoglossicida* was constructed by using the red recombination system (Wang et al., 2017). According to the reference genomic sequence of *P. plecoglossicida* NZBD9, the upstream and downstream homologous sequences of *fusA* were searched, and each 20 bp before and after the gene was selected as the homologous fragments of primers, and the upstream and downstream primer sequences of the tetracycline resistance gene of plasmid pCM130 were amplified by fusion at the 3′ end of primers. After successful amplification, tetracycline-resistant gene fragments were recovered by 1% gel electrophoresis and DNA fragment recovery kit. The plasmid pKD46 was transformed into *P. plecoglossicida* NZBD9 by electroporation and cultured into OD_600_ = 0.2–0.3. The previously recovered fragment of the 10-μl resistance gene was transferred into *P. plecoglossicida* with pKD46 by electroporation, and L-arabinose of 10–30 mmol/l was added, so that the recombinant enzymes Exo, Bet, and Gam of pkD46 were fully expressed (Wang et al., 2017). The mutant bacteria were cultured overnight in LB plates (containing 100 μg/ml tetracycline) at 28°C. The positive colonies were validated by polymerase chain reaction (PCR) and gene sequencing.

### 2.4 Growth Curve of *fusA* Mutant

The *P. plecoglossicida fusA* knockout strain was cultured at 28°C (OD_600_ = 0.3). The wild-type strain of *P. plecoglossicida* was used as the control. OD_600_ were recorded once an hour for a total of 24 h, and growth curves of wild-type and mutant strains were compared. Three independent biological replications were performed for each data point.

### 2.5 Semisolid Agar Plate Motility Assay

The semisolid agar method was used to measure the motility of *P. plecoglossicida* (Zhang et al., 2018b). In short, the mutants and wild type of *P. plecoglossicida* were cultured overnight at 28°C (220 rpm) and adjusted to an OD_600_ of 0.3. Firstly, the sterilized toothpicks were immersed in the processed bacterial solution, and then immersed in the center of a semi-solid agar plate (LB broth + 0.5% agar), and finally incubated at 28°C for 20 h. The colony diameter was measured by the instrument, each in three independent biological replicates.

### 2.6 Biofilm Assay

The biofilm assay for *P. plecoglossicida* was carried out as described by Huang et al. (2021). Firstly, the mutants and wild-type strains of *P. plecoglossicida* were cultured overnight at 28°C, and then the OD_600_ of the culture was adjusted to 0.2. 150 μl LB was mixed with 50 μl bacterial culture medium and then incubated at 28°C for 24 h, washed with aseptic PBS for 3 times, stained with 200 μl 11% crystal violet for 15 min, then rinsed with aseptic PBS and air dried. The biofilm was dissolved with 200 μl of acetic acid (33%) and quantified by OD_590_. Six independent biological replications were performed for each group.

### 2.7 Adhesion Assay *In Vitro*

An adhesion assay of *P. plecoglossicida* was carried out *in vitro* by the microscope counting method (Rh et al., 2020). First, 20 μl of sterile large yellow croaker epidermis mucus was added to the center of clean glass slides, then it was spread evenly with a coverslip and incubated overnight, then fixed with 4% methanol solution at room temperature for 30 min and centrifuged to collect the mutants and wild strains of *P. plecoglossicida* cultured overnight, and finally resuspended in PBS. According to the value of OD_560_, the suspension was adjusted to 10^8^ CFU/ml. 200 μl of bacterial solution was added to the slides and incubated at 28°C for 2 h, rinsed repeatedly with sterile PBS for 5 times, and then air dried, and fixed with 4% methanol solution at room temperature for 30 min. Finally, the slides were dyed with 1% crystal violet for 3 min and then observed with a microscope and imaged with a digital camera (magnification, ×1,000). The number of bacteria was quantified from the image using IPwin software (3 slides with 20 visual fields per slide) (Ryckaert et al., 2010; Isla et al., 2014; Zhang et al., 2018b; Huang et al., 2020a).

### 2.8 Hemolysis Assay

The hemolysis assay for *P. plecoglossicida* was carried out as described by Wei et al. (Tsou and Zhu, 2010; Xu et al., 2021). 100 μl of fresh sheep blood (Ping Rui Biotechnology Co., Ltd., Beijing, China) was centrifuged at 2,500 rpm for 5 min, and the supernatant was discarded. The red blood cells were then rinsed with 200 μl PBS three times and then resuspended with 100 μl PBS. The mixture of 5 μl of resuspended red blood cells with 245 μl of wild type or *P. plecoglossicida* Δ*fusA* culture was incubated at 37°C (150 rpm) for 1 h. Finally, the mixture was centrifuged at 5,000 rpm at room temperature for 3 min, and then 100 μl of the supernatant was used to determine the OD_540_.

### 2.9 Total RNA Extraction and Reverse Transcription

TRIzol reagent (Invitrogen, Carlsbad, CA, USA) was used for total RNA extraction from bacterial cells, as directed by the manufacturer. Reverse transcription was carried out with A Reverse Aid Mu-MLV cDNA synthesis kit (TransGen Biotech Co., Ltd., Beijing, China) from 2.0 mg total RNA, as instructed by the manufacturer (Ge et al., 2020; Huang et al., 2020a; Xu et al., 2021).

### 2.10 qRT-PCR

qRT-PCR adopts the SYBR Green method and refers to the TransStart Top Green qPCR SuperMix kit instructions. **Table 1** shows all the sequences used in the experiment. The reaction system is 10 μl, containing 0.5 μl template, 0.25 μl forward primer (10 μM), 0.25 μl reverse primer (10 μM), and 9.5 μl 2× TransStart Top Green qPCR SuperMix, and then amplified and detected on the QuantStudio™ 6 Flex real-time fluorescent quantitative PCR system instrument. Finally, the 2^-ΔΔCt^ calculation method was used to calculate the relative expression level of genes, while gene expression levels were normalized to 16S RNA (Feng et al., 2016; Ge et al., 2020).

**Table 1:** Primers for qRT-PCR.

Genes	Primers
*fusA-Ⅰ*	F: 5′-CACAGGTCTGAGCCACAAG-3′
	R: 5′-CATACGGTTTCGGACTGC-3′
*fusA-Ⅱ*	F: 5′-ATGGCTCGTACAAGCAATTAACC-3′
	R: 5′-TTAGCTTGTTATAACGGTGCT-3′
Δ*fusA*	F: 5′-ATGGCTCGTACTACAGCAATGTGAAACCCAACATACCCCTGATC-3′
	R: 5′-AATCGGAACAAAAAATTGCTTCAGCGATCGGCTCGTTGC-3′
*16S-rRNA*	F: 5′-GTTGGGAGGAAGGGCAGTAAG-3′
	R: 5′-ATCTAGGCATTTCACCGCTACA-3′

fusA-Ⅰ is the sequence used in qRT PCR, fusA-Ⅱ is the full field verification sequence of the fusA gene, and ΔfusA is the knockout verification sequence of the fusA gene.

### 2.11 Statistical Analysis

Data were presented as mean ± standard deviation (SD) and analyzed by SPSS 18.0 software (IBM, Armonk, NY, USA). Differences were compared by one-way analysis of variance (ANOVA) followed by the Dunnett’s test. *p* < 0.05 was considered statistically significant.

## 3 Results

### 3.1 Genetic Evolution and Protein Structure Analysis of *fusA* of *P. plecoglossicida*

**Figure 1:**
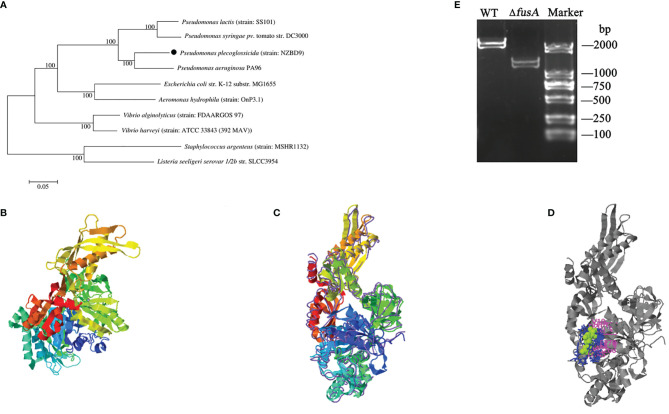
Phylogenetic tree and predicted protein structure of *fusA* of *P. plecoglossicida* and PCR identification of *P. plecoglossicida* Δ*fusA*. Note: **(A)** is the phylogenetic tree of *fusA of P. plecoglossicida.* MEGA7 is used to construct the adjacent tree. The self-development value is set to 1,000 times, and the self-development value ≥60% is displayed on the main nodes. The isolates in this experiment are represented by solid circle; **(B)** is the predicted *P. plecoglossicida* FusA protein structure model; **(C)** is the closest *P. plecoglossicida* FusA protein structure model to the known protein structural model in PDB; **(D)** is active sites of the *P. plecoglossicida* FusA protein structure model; **(E)** is the PCR identification of *P. plecoglossicida* Δ*fusA* (primers: Δ*fusA*-F and Δ*fusA*-R). Maker: DS2000 DNA marker; WT: PCR amplifications with wild-type genomic DNA (2,148 bp); Δ*fusA*: PCR amplifications with Δ*fusA* genomic DNA (1,204 bp).

**Supplemental 1** shows the comparison result of the FusA amino acid sequences of *P. plecoglossicida* (NZBD9) and nine pathogenic bacteria. From the comparison results, it can be seen that the similarity between FusA amino acid sequences of *P. plecoglossicida* (NZBD9) (f) and *P. aeruginosa* PA96 (j) was relatively high. The phylogenetic tree analysis of *P. plecoglossicida* (NZBD9) and the other nine pathogenic bacteria *fusA* showed that *P. plecoglossicida* (NZBD9) and *P. aeruginosa* PA96 (j), *P. syringae pv.* tomato str. DC3000 (g), and *P. lactis* (strain: SS101) (d) had the closest genetic distance (**Figure 1A**). The protein structure was predicted by I-TASSER: among the five largest structural clusters, we selected the one with the highest confidence score, namely, C-score = 0.16 (**Figure 1B**). According to the highest TM score, the protein with the closest structural similarity to the predicted protein model in PDB was selected (PDB ID: 2XEX) (**Figure 1C**). The active site analysis also yielded the prediction result with the highest confidence (C-score = 0.60, **Figure 1D**). The closest *P. plecoglossicida* FusA protein structure model to the known protein structural model in PDB was *Staphylococcus aureus*. As we all know, proteins with high structural similarity often have similar function to the target. The predicted *P. plecoglossicida* FusA protein structure was biologically annotated by COFACTOR and COACH; the protein function and the conservation of the active site were inferred. It was found that the FusA protein function active site was highly conserved.

### 3.2 Construction of the Δ*fusA* Mutant of *P. plecoglossicida*

The target fragment of *fusA* amplified by PCR was introduced into *P. plecoglossicida* NZBD9 by electrical transformation, and the *fusA* gene was knocked out by the λ-red recombination system. The result was confirmed by a PCR identification (**Figure 1E**) and DNA sequencing (data not shown), verifying that a knockout mutant of Δ*fusA* was successfully constructed.

### 3.3 The Effect of the *fusA* Gene on the Growth of *P. plecoglossicida*

**Figure 2:**
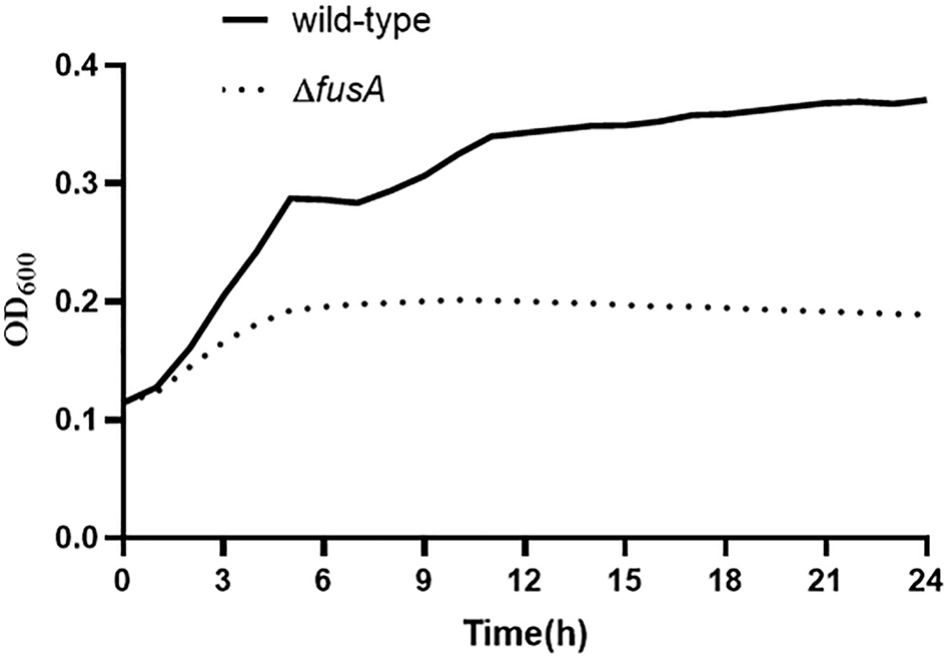
Determination of growth ability of wild-type and Δ*fusA* of *P. plecoglossicida*.

In order to evaluate the difference of growth ability between Δ*fusA* and the wild type of *P. plecoglossicida*, the 24-h growth curves of wild-type *P. plecoglossicida* NZBD9 and *ΔfusA* at 28°C were compared. Through the 24-h growth curve test, the results showed that the growth curves of the two strains were significantly different, and the differences between sampling points were significant (*p* < 0.05) (**Figure 2**). The wild type and Δ*fusA* had no significant difference at the first 2-h adaptation period, but at the other similar time points, the OD_600_ of the Δ*fusA* growth curve was significantly lower than that of the wild type, indicating that the growth rate of the mutant strain was significantly lower than that of the wild strain. According to our results, the expression of *fusA* has a significant impact on the growth of *P. plecoglossicida*, especially in the middle to later stages of growth.

### 3.4 Effect of the *fusA* Gene on Motility of *P. plecoglossicida*

**Figure 3:**
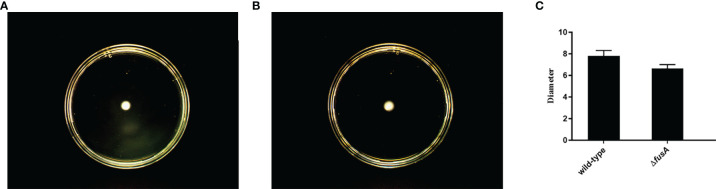
Determination of colony motility of wild-type and Δ*fusA* of *P. plecoglossicida*. **(A)** is the plate movement diagram of the wild-type of *P. plecoglossicida*; **(B)** is the plate movement diagram of the Δ*fusA* colony of *P. plecoglossicida*; **(C)** is the colony diameter histogram.

Flagella are a special structure of bacteria. Bacteria rely on the flagellum to achieve movement ability. The motility of bacteria has a direct impact on the chemotaxis of bacteria, which helps to move to a suitable environment and colonize the host. It is an important virulence factor of pathogenic bacteria. In order to detect whether the *fusA* gene affects the motility of *P. plecoglossicida*, the motility of the wild type and Δ*fusA* of *P. plecoglossicida* was tested. The results showed (**Figure 3**) that the colony diameters of the wild type and Δ*fusA* of *P. plecoglossicida* placed on semisolid agar plates for 20 h were about 8.119 ± 0.66 mm and 8.359 ± 0.49 mm. Statistical analysis showed that there was no significant difference between the two strains (*p* > 0.05). Therefore, it can be concluded that the *fusA* gene does not play a significant role in bacterial movement.

### 3.5 Effect of the *fusA* Gene on the Biofilm-Forming Ability of *P. plecoglossicida*

**Figure 4:**
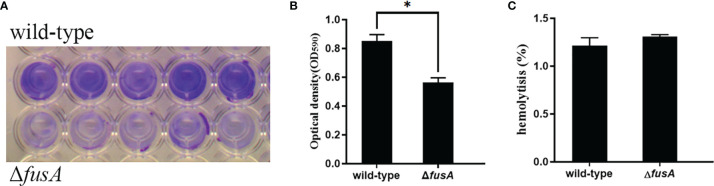
Determination of the biofilm formation and hemolytic ability of wild-type and Δ*fusA* of *P. plecoglossicida*. **(A)** is the typical figure of the biofilm formation of the wild type and Δ*fusA*; **(B)** is the biofilm formation histogram of the wild-type and Δ*fusA*; **(C)** is the hemolytic ability histogram of wild-type and Δ*fusA*; *P* < 0.05 is marked as *.

The bacteria with biofilm are highly resistant to antibiotics, and the biofilm can reinfect the host by releasing bacteria in the biofilm. Once the biofilm is formed, it is difficult to eradicate. In order to prove whether the *fusA* gene is related to biofilm formation, the biofilm formation of Δ*fusA* and the wild type of *P. plecoglossicida* was detected by OD_590_ nm (**Figure 4**). The results showed that the biofilm-forming ability of Δ*fusA* was lower than that of the wild type. It can be concluded that the *fusA* gene has a certain effect on the biofilm formation of *P. plecoglossicida*.

### 3.6 Effect of the *fusA* Gene on the Adhesion Ability of *P. plecoglossicida*

**Figure 5:**
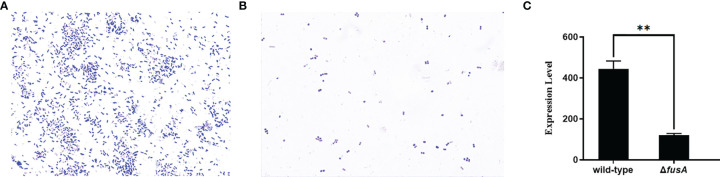
Determination of adhesion ability of wild type and Δ*fusA* of *P. plecoglossicida.* **(A)** is the typical vision of the adhesion of wild type under the optical microscope; **(B)** is the typical vision of the adhesion of Δ*fusA* under the optical microscope; **(C)** is the histogram of the number of bacteria in a field of the optical microscope; *p* < 0.01 is marked as **.

Adhesion is one of the important factors for pathogenic bacteria to invade the host. In order to verify whether the *fusA* gene was related to bacterial adhesion, the adhesion of the wild type and *fusA* mutant strains to the mucus was observed under the microscope, and the number of bacteria was quantified from the image with IPwin software (**Figure 5**). It could be seen from the figure that the adhesion ability of Δ*fusA* was significantly lower than that of the wild type, indicating that *fusA* was involved in the regulation of adhesion.

### 3.7 Effect of the *fusA* Gene on the Hemolytic Ability of *P. plecoglossicida*

Hemolysin is also one of the important virulence factors of bacteria. In order to verify whether the *fusA* gene was involved in the hemolysis mechanism of *P. plecoglossicida*, the hemolysis assay was carried out. The results showed (**Figure 4C**) that the hemolytic ability of Δ*fusA* was not significantly different from that of the wild type. This indicated that the *fusA* gene might not participate in the hemolysis process of *P. plecoglossicida.*

### 3.8 Verification of *fusA* Expression Under Different Stress Environments

#### 3.8.1 The Expression Level of *fusA* at Different Temperatures

**Figure 6:**
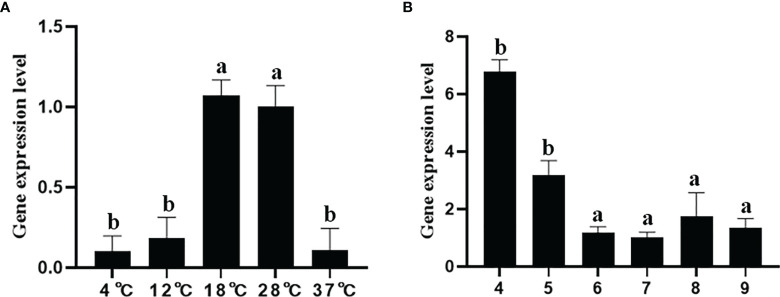
The expression level of the *fusA* gene under different temperature stresses **(A)** and different pH stresses **(B)**. The means of the treatments not sharing a common letter are significantly different at *p* < 0.01.

Temperature is one of the chief environmental factors that affect bacterial survival inside or outside their hosts, especially *P. plecoglossicida* which is a temperature-dependent pathogen. Therefore, qRT-RCR was used to detect the expression level of *fusA* at 4°C, 12°C, 18°C, 28°C, and 37°C (**Figure 6A**). The results showed that the expression level of *fusA* at 4°C, 12°C, and 37°C was significantly lower than that at 18°C and 28°C; the expression level of *fusA* at 18°C has no significant difference compared with that at 28°C. 18°C is the pathogenic temperature of *P. plecoglossicida*. It can be seen that *fusA* may be involved in the temperature-related virulence regulation of *P. plecoglossicida.*

#### 3.8.2 The Expression Level of *fusA* at Different pH

pH is an important environmental factor that restricts the survival and growth of bacteria, which was usually used to defend the bacterial infection by hosts. Therefore, qRT-PCR was used to detect the expression of *fusA* at pH = 4, 5, 6, 7, 8, and 9. The results showed that (**Figure 6B**), compared with the pH = 7 group, the expression of *fusA* was significantly higher at pH = 4 and 5, and there was no significant difference between the other groups and the pH = 7 group.

#### 3.8.3 The Expression Level of *fusA* Under Stresses of Heavy Metal Ion

**Figure 7:**
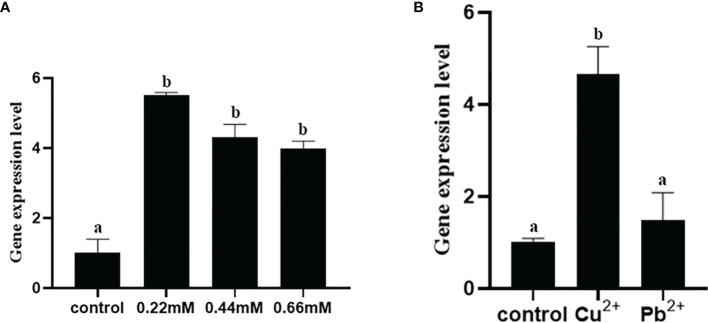
The expression level of *fusA* gene under heavy metal ion stress **(A)** and different concentrations of H_2_O_2_ **(B)**. The means of the treatments not sharing a common letter are significantly different at *p* < 0.01.

qRT-PCR was used to detect the expression of *fusA* under Cu^2+^ and Pb^2+^ stress. The results show that (**Figure 7A**) compared with the control group, the expression of *fusA* increased significantly under Cu^2+^ stress, while under Pb^2+^ stress, there is no significant difference, which means that Cu^2+^ may have an inducing effect on the expression of *fusA*, while Pb^2+^ has no obvious effect.

#### 3.8.4 The Expression Level of *fusA* Under Different Concentrations of H_2_O_2_

qRT-PCR was used to verify the expression of *fusA* at 0.22, 0.44, and 0.66 mmol/l H_2_O_2_. The results showed (**Figure 7B**) that the expression of *fusA* increased significantly under the stress of three H_2_O_2_ concentrations, but there was no significant difference in the expression of *fusA* among various concentrations. This indicated that H_2_O_2_ could promote the expression of *fusA*.

### 3.9 Expression Level of *fusA* in Iron-Poor Environment

**Figure 8:**
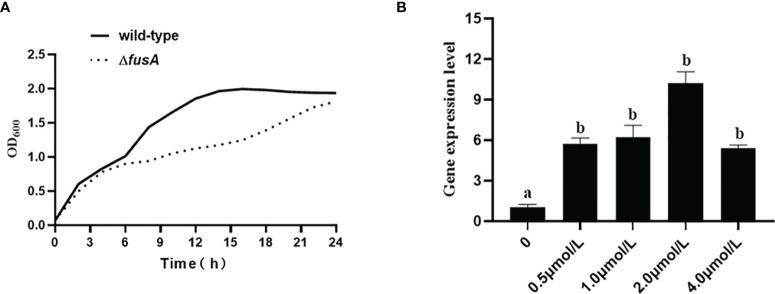
Determination the function of *fusA* in iron acquisition. **(A)** displays the growth ability of wild type and Δ*fusA* of *P. plecoglossicida* in the concentration of 2,2-bipyridine at 1 μmol/l; **(B)** is a histogram of *fusA* gene expression levels under the treatment of 2,2-bipyridine at 0.5, 1, 2, and 4 μmol/l, respectively. The means of the treatments not sharing a common letter are significantly different at *p* < 0.01.

As a member of the TBDR family, *fusA* is indispensable in the transport of ferredoxin. In order to verify the function of *fusA* in an iron-poor environment, growth assay and qRT-PCR were carried out. The results showed (**Figure 8**) that the growth of the Δ*fusA* strain in the iron-poor environment decreased significantly compared with the wild-type strain, indicating that the *fusA* gene might be involved in the utilization of Fe^2+^ by *P. plecoglossicida*. Meanwhile, compared with the control group, when the concentration of 2,2-bipyridine is 0.5, 1, 2, and 4 μmol/l, the *fusA* gene expression level was significantly upregulated, and its expression reaches the maximum value at 2 μmol/l.

## 4 Discussion

FusA amino acid sequence alignment and phylogenetic tree displayed that *P. plecoglossicida* has a high degree of homology with *P. aeruginosa*, which indicated the biological importance of FusA among *Pseudomonas* (Feng et al., 2016; Jones et al., 2017). Prediction of the *P. plecoglossicida* FusA protein structure model was carried out by I-TASSER (Yang and Zhang, 2015; Zhang et al., 2017; Zheng et al., 2021). The closest *P. plecoglossicida* FusA protein structure model to the known protein structural model in PDB was *Staphylococcus aureus*. As we all know, proteins with high structural similarity often have similar function to the target. The predicted *P. plecoglossicida* FusA protein structure was biologically annotated by COFACTOR and COACH; the protein function and the conservation of the active site were inferred. It was found that the FusA protein function active site was highly conserved.

All living organisms cannot do without trace metal nutrients, such as transition metals like iron (Fe), manganese (Mn), zinc (Zn), and molybdenum (Mo) (Radin et al., 2016; Radin et al., 2019a). According to proteomics or bioinformatics analysis, 30% of proteins in organisms need to interact with metal ions (Grim et al., 2017; Andreini et al., 2008; Waldron and Robinson, 2009; Waldron et al., 2009). During the infection, the pathogen’s nutrients all come from the host, and the host restricts the supply of necessary trace metals in order to resist the invasion of the pathogen (Weinberg, 2009; Kehl-Fie and Skaar, 2010), so that the pathogen is in a metal starvation state, that is, nutritional immunity (Corbin et al., 2008; Kehl-Fie and Skaar, 2010; Hood and Skaar, 2012; Huang et al., 2021). The most characteristic inhibitory response of nutritional immunity is iron (Schaible and Kaufmann, 2004; Hood and Skaar, 2012; Párraga Solórzano et al., 2019; Radin et al., 2019b).

For all living organisms, iron is an important element. Bacteria have also evolved ways to obtain iron. TBDRs are an active transporter on the outer membrane of Gram-negative bacteria (Lim, 2010; Noinaj et al., 2010; Fujita et al., 2019), which use the energy produced by the inner membrane to transport nutrients (such as sugars, etc.) through the TonB/ExbBD complex (Saier, 2000; Nikaido, 2003; Braun, 2006; Schauer et al., 2008; Jordan et al., 2013; Modrak et al., 2018). *fusA* is a newly discovered member of the TBDR family, which is also involved in iron transport. With the continuous study of TBDRs, it has been found that TBDR is also involved in host infection. Many laboratories have previously reported that various factors in the TBDR family are involved in virulence regulations in different bacteria. For example, in *Flavobacterium psychrophilum*, the *exbD* loci of a TonB system are required for optimal bacterial virulence (Álvarez et al., 2008), and *tdrA* in TBDRs is also an indispensable virulence factor in the pathogenicity of *Pseudomonas fluorescens* (Hu et al., 2012). According to our genomic analysis, the *fusA* gene existed in the *P. plecoglossicida* genome. In the previous dual RNA-seq of our laboratory, the *fusA* gene was significantly highly expressed, indicating that it may be an important virulence gene of *P. plecoglossicida*. In view of the importance of *fusA* in other bacterial pathogens, there is no indication that *fusA* is involved in the virulence regulation of *P. plecoglossicida* previously reported, and it is also not clear which pathogenic factors this gene is related to in *P. plecoglossicida*, so we investigated the relationship between *fusA* and the pathogenicity of *P. plecoglossicida*. In the present study, the *P. plecoglossicida fusA* gene knockout strain was successfully constructed, and the virulence phenotype of the mutant strain was determined.

Mucus is abundant on the surface of fish skin, gill, and intestinal wall, which provides a good adhesion environment for bacteria. Therefore, it is the first site where pathogen and host are most likely to interact (Liu et al., 2020). After the bacteria adhere to the host, they may begin to invade, and in the process, the bacteria will try to protect themselves from the host’s immune system. For example, the formation of biofilm is one of the measures to resist host immune attack (Qin et al., 2016). The results showed that there were significant differences in adhesion and bacterial growth rate between the wild type and Δ*fusA* of *P. plecoglossicida*. The absence of the *fusA* gene will weaken the adhesion of bacteria and the ability of tissue transmission and colonization. The TBDR family is known to be important in bacterial uptake of trace iron (Wang et al., 2016), and *fusA* is a member of TBDRs. In the iron-poor environment, the *fusA* expression level was dramatically upregulated, indicating that the *fusA* gene played an important role in the competition of *P. plecoglossicida* for the trace iron-related virulence mechanism in harsh environments. Flagella are not only a motility organ of bacteria but also one of the parameters for evaluating the virulence factors of pathogenic bacteria (Nakamura and Minamino, 2019). Relevant studies have shown that flagella are not only involved in bacterial motility but also involved in bacterial adhesion, biofilm formation, and special channel transport of virulence proteins and other pathways, which indirectly affect bacterial virulence (Feldman et al., 1998; Blocker et al., 2003; Roy et al., 2009; Duan et al., 2013; Duan et al., 2013). Although the motility and hemolytic ability of the *P. plecoglossicida ΔfusA* were not different from the wild type, the biofilm formation ability and adhesion ability of Δ*fusA* were significantly reduced. Taken together, *fusA* was obviously involved in the virulence regulation of *P. plecoglossicida.*

Bacterial virulence is usually affected by environmental factors. For example, the pathogenic temperature of “visceral white spot disease” caused by *P. plecoglossicida* in large yellow croaker and other commercial fish is 15°C–20°C (Zhang et al., 2018a; Kaur et al., 2019; Lv et al., 2019; Huang et al., 2020a; Hu et al., 2021; Jin et al., 2021). The results of various stress experiments showed that compared with 18°C, the expression level of *fusA* was significantly downregulated at 4°C, 12°C, and 37°C. This indicated that *fusA* may be involved in the temperature-related virulence regulation of *P. plecoglossicida.* Besides, the expression level was significantly upregulated under acidic conditions as pH = 4 or 5, which indicated that *fusA* was involved in the response to low pH. Meanwhile, the significantly high expression of *fusA* under Cu^2+^ exposure and H_2_O_2_ conditions also further indicated that *fusA* was important for *P. plecoglossicida* survival under severe conditions.

In conclusion, through *fusA* mutant strain construction, we found that the *fusA* gene was involved in a variety of pathogenic and environmental adaptation mechanisms of *P. plecoglossicida*. At the same time, whether *fusA* existed or not could promote *P. plecoglossicida* to compete for iron in the environment. These results further confirmed the importance of *P. plecoglossicida fusA* and laid a foundation for further study on the function of the gene.

## Data Availability Statement

The original contributions presented in the study are included in the article/**Supplementary Material**. Further inquiries can be directed to the corresponding authors.

## Author Contributions

All authors contributed to the article and approved the submitted version. QY and LH conceived the experiments. RH, JW, ML, JT, BW, XT, JZhou, and JZhang conducted the experiments. All authors assisted in the collection and interpretation of data. LH and QY wrote the manuscript. All authors contributed to the article and approved the submitted version.

## Funding

This work was supported by grants from the major program of Science and Technology Planning of Xiamen under Contract No. 3502Z20211004 and the Fujian Engineering Research Center of Aquatic Breeding and Healthy Aquaculture, under Contract No. DF201903.

## Conflict of Interest

The authors declare that the research was conducted in the absence of any commercial or financial relationships that could be construed as a potential conflict of interest.

## Publisher’s Note

All claims expressed in this article are solely those of the authors and do not necessarily represent those of their affiliated organizations, or those of the publisher, the editors and the reviewers. Any product that may be evaluated in this article, or claim that may be made by its manufacturer, is not guaranteed or endorsed by the publisher.

## Supplementary Material

Supplemental Figure 1 The comparison result of the *fusA* amino acid sequences of *P. plecoglossicida* (NZBD9) and nine pathogenic bacteria. Note: The dark background sequence indicates the same *fusA* amino acid sequence of *P. plecoglossicida* NZBD9 as the other nine pathogenic bacteria.
