# Supplementary material for: Effect of Ferredoxin Receptor FusA on the Virulence Mechanism of Pseudomonas plecoglossicida
Source: Front Cell Infect Microbiol. 2022 Mar 22;12:808800. doi: 10.3389/fcimb.2022.808800 (PMC8981516; doi:10.3389/fcimb.2022.808800)
Supplement: Supplemental Figure 1 — The comparison result of the fusA amino acid sequences of P. plecoglossicida (NZBD9) and nine pathogenic bacteria. Note: The dark background sequence indicates the same fusA amino acid sequence of P. plecoglossicida NZBD9 as the other nine pathogenic bacteria. [file Image_1.pdf]

```
  1        10        20        30        40        50        60
a MARTTPIARYRNIGISAHIDAGKTTTTERILFYTGVNHKIGEVHDGAATMDWMEQEQERG
i MARTTPIERYRNIGISAHIDAGKTTTTERVLFYTGVSHKIGEVHDGAATMDWMEQEQERG
c .................................................MDWMEQEQERG
h .................................................MDWMEQEQERG
d MARTTPISRYRNIGIVAHVDAGKTTTTERVLFYTGKSHKMGEVHDGAATTDWMVQEQERG
g MARTTPISRYRNIGIVAHVDAGKTTTTERVLFYTGKSHKMGEVHDGAATTDWMVQEQERG
f MARTTAINRYRNIGICAHVDAGKTTTTERILFYTGLSHKMGEVHDGAATTDWMVQEQERG
j ....................................................MVQEQERG
b MAREFSLEKTRNIGIMAHIDAGKTTTTERILYYTGRIHKIGETHEGASQMDWMEQEQDRG
e MAREFSLEKTRNIGIMAHIDAGKTTTTERILFYTGRIHKIGETHEGASQMDWMEQEQERG

           70        80          90        100       110
a ITITSAATTAFWSGMAKQYEPHR.INIIDTPGHVDFTIEVERSMRVLDGAVMVYCAVGGV
i ITITSAATTAFWSGMGKQFQPHR.INIIDTPGHVDFTIEVERSMRVLDGAVMVYCAVGGV
c ITITSAATTTFWRGMEAQFPEHR.VNIIDTPGHVDFTIEVERSLRVLDGAVVVFCGSSGV
h ITITSAATTTFWRGMEAQFQEHR.VNIIDTPGHVDFTIEVERSLRVLDGAVVVFCGSSGV
d ITITSAAITAFWKGSEKQYKDEHRFNVIDTPGHVDFTIEVERSLRVLDGAVVVFCGTSGV
g ITITSAAITAFWQGSEKQHKDQYRFNVIDTPGHVDFTIEVERSLRVLDGAVVVFCGTSGV
f ITITSAAVTTFWKGS.RGQYDNYRVNVIDTPGHVDFTIEVERSLRVLDGAVVVFCGTSGV
j ITITSAAVTTFWKGS.RGQYDNYRVNVIDTPGHVDFTIEVERSLRVLDGAVVVFCGTSGV
b ITITSAATTAAWEGH........RVNIIDTPGHVDFTVEVERSLRVLDGAVTVLDAQSGV
e ITITSAATTAQWKGY........RVNIIDTPGHVDFTVEVERSLRVLDGAVAVLDAQSGV

120       130       140       150       160       170
a QPQSETVWRQANKYKVPRIAFVNKMDRMGANFLKVVNQIKTRLGANPVPLQLAIGAEEHF
i QPQSETVWRQANKYKVPRIAFVNKMDRTGANFLRCVEHIKTRLKGNPVPLQLNIGSEENF
c EPQSETVWRQADKYHVPRLVFVNKMDRAGADFLRVVNQIKNRLGATPVPIQLNVGAEDEF
h EPQSETVWRQADKYHVPRMVFVNKMDRAGADFLRVVDQIKNRLGANPVPIQLNVGAEDDF
d EPQSETVWRQANKYGVPRLVYVNKMDRAGANFLRVIGQIKQRLGHTPVPIQLAIGSEDNF
g EPQSETVWRQANKYGVPRIVYVNKMDRAGANFLRVIAQIKQRLGHTPVPIQLAIGAEDNF
f EPQSETVWRQANKYGVPRVVYVNKMDRAGANFLRVVGQIKNRLGHTPVPVQLAIGAEDDF
j EPQSETVWRQANKYGVPRIVYVNKMDRQGANFLRVVEQIKKRLGHTPVPVQLAIGAEENF
b EPQTETVWRQATTYGVPRIVFVNKMDKLGANFEYSVSTLHDRLQANAAPIQLPIGAEDEF
e EPQTETVWRQATTYGVPRVVFVNKMDKIGADFLYSVGTLHDRLAANAHPIQLPIGAEDTF

180       190       200       210       220       230
a TGVVDLVKMKAINWNDADQGVTFEYEDIPADMVELANEWHQNLIESAAEASEELMEKYLG
i KGVIDLVKMKAINWSEADQGVSFDYEDVPAELLEKAQEMRMTLVEAAAEASEDLMEKYLG
c RGVIDLIKMKMINWNDADQGTSFTYEDIPADMIELAEEWRNHMVESAAEASEELMDKYLE
h RGVIDLIKMKMINWNEADQGMSFTYDEIPADMQELAEEWRNNLVEAAAEATEELMDKYLE
d QGQIDLINMEAVYWNDSDKGMVPVRKPIPEELQELADEWRNNMVEAAAEASEELMNKYLE
g QGQIDLMAMEAVYWNDSDKGMVPVRKPIPAELQELADEWRGNMVEAAAEASEELMNKYLE
f QGQVDLIKMKAIYWNDDDKGTTYREEEIPADMLDLAEEWRSNMVEAAAEANEELMNKYLE
j VGQVDLIKMKAIYWNDDDKGMTYREEEIPAELKDLAEEWRSSMVEAAAEANEELMNKYLE
b EAIIDLVEMKCFKYTN.DLGTEIEEIEIPEDHLDRAEEARASLIEAVAETSDELMEKYLG
e EGIIDLIEMNALYYED.DLGNDPHIKEIPADLKELADEYRGKLVEAVAELDEELMMKYLE

240       250       260       270       280       290
a GEELTEAEIKGALRQRVLNNEIILVTCGSAFKNKGVQAMLDAVIDYLPSPVDVPAINGIL
i GEELTEEEIKKALRQRVLNNEIILVTCGSAFKNKGVQAMLDAVVDYLPAPTDVAAIDGLK
c EGELTEAELKQALRTRTLNNEIVLATCGSAFKNKGVQAVLDAVVEFLPSPVDVPAIKGVD
h EGELSEAEIKQALRTRTLNNEIVLATCGSAFKNKGVQAVLDAVIEFLPSPIDVPAIKGID
d GEELTNVEIKAALRQRTIAGEIVLAVCGSSFKNKGVPLVLDAVIDYLPAPTDIPAIKGSN
g GEELTNEEIKAALRQRTIAGEIVLAVCGSSFKNKGVPLVLDAVIDYLPAPIDIPAIKGSD
f EGDLSVEDIKAGLRARTLASEIVPAVCGSSFKNKGVPLVLDAVIDFLPAPTEIPAIKGIH
j EGELSEAEIKEGLRLRTLACEIVPAVCGSSFKNKGVPLVLDAVIDYLPAPTEIPAIKGVS
b DEEISVAELKEAIRQATTNVEFYPVLCGTAFKNKGVQLMLDAVIDYLPSPLDVKPIIGHR
e GEEITKEELKAGIRKGTLDVEFYPVVCGTAFKNKGVQPMLDAVLDYLPAPTDVPAINGVL

300                310       320       330       340       350
a DDGKDTPAER.........HASDDEPFSALAFKIATDPFVGNLTFFRVYSGVVNSGDTVL
i LDG.ETKDER.........HASDDEPFAALAFKIATDPFVGNLTFFRVYSGVVNSGDSVL
c ERE..NEVER.........HADDNEPFSALAFKIATDPFVGTLTFMRVYSGVVNSGDAVY
h ERE..NEVER.........HADDNEPFSALAFKIATDPFVGTLTFMRVYSGVVNSGDAVY
d PDNEEEE.........MERHADDNEPFSALAFKIATDPFVGTLTFVRVYSGVLASGDGVI
g PDNEEIL.........MERHADDSEPFSALAFKIATDPFVGTLTFVRVYSGVLASGDGVI
f PDLIDVPKDEVTPEQYDERPADDNEPFSALAFKIATDPFVGTLTFVRVYSGFLTSGDSVI
j PDDETVE.........DERHADDNEPFSSLAFKIATDPFVGTLTFARVYSGVLSSGDSVL
b ASNPEEEVIA.........KADDSAEFAALAFKVMTDPYVGKLTFFRVYSGTMTSGSYVK
e PDG..EEAAR.........HADDSEPFSSLAFKVMTDPYVGRLTFFRVYSGTLNSGSYVQ
```

```
               360       370       380       390       400       410
a   NSVKAARERFGRIVQMHANKREEIKEVRAGDIAAAIGLKDVTTGDTLCDPDAPIILERME
i   NSVKEKRERFGRIVQMHANKREEIKEVRAGDIAAAIGLKDVTTGDTLCDEKAPIILERME
c   NSVKEKKERFGRIVQMHANKREEVKEVRAGDIAAAIGLKDVTTGDTLCDQNHKVILERME
h   NSVKDKKERFGRIVQMHANKREEVKEVRAGDIAAAIGLKDVTTGDTLCDQNHKVILERME
d   NSVKGKKERVGRMVQMHANAREEIKEVRAGDIAALIGMKDVTTGETLCDAAKPIILVRMD
g   NSVKGKKERVGRMVQMHANAREEIKEVRAGDIAALIGMKDVTTGETLCNADKPIILVRMD
f   NSVKGKKERVGRMVQMHANQREEIKEVRAGDIAALIGMKDVTTGDTLCNADKPIILERMD
j   NSVKGKKERVGRMVQMHANQREEIKEVRAGDIAALIGMKDVTTGDTLCSIEKPIILERMD
b   NSTKGKRERVGRLLQMHANSRQEIDTVYSGDIAAAVGLKDTGTGDTLCGEKNDIILESME
e   NSTKGKRERVGRILQMHANHREEISIVYAGDIAAAVGLKDTTTGDTLCDEKDQIILESME
```

```
               420       430       440       450       460       470
a   FPEPVISIAVEPKTKADQEKMGLALGRLAKEDPSFRVWTDEESNQTIIAGMGELHLDIIV
i   FPEPVISIAVEPKTKADQEKMGLALGRLAQEDPSFRVWTDEESGQTIIAGMGELHLDIIV
c   FPEPVIQIAVEPRSKADQEKMAIALGKLAAEDPSFRVETDDESGQTLISGMGELHLDIIV
h   FPEPVIQIAVEPRSKADQEKMGIALGKLAAEDPSFRVETDDETGQTLISGMGELHLDIIV
d   FPEPVISVAVEPKTKDDQEKMGIALGKLAQEDPSFRVKTDEETGQTIISGMGELHLDILV
g   FPEPVISVAVEPKTKDDQEKMGIALGKLAQEDPSFRVKTDEETGQTIISGMGELHLDILV
f   FPEPVISLSVEPKTKADQEKMGIALGKLAQEDPSFRVKTDEETGQTIISGMGELHLDILV
j   FPEPVISVAVEPKTKADQEKMGIALGKLAQEDPSFRVKTDEESGQTIISGMGELHLDIIV
b   FPEPVIHLSVEPKSKADQDKMTQALVKLQEEDPTFHAHTDEETGQVIIGGMGELHLDILV
e   FPEPVIQVAIEPKSKADQDKMGQALAKLAEEDPTFRAETDQETGQTLISGMGELHLDILV
```

```
               480       490       500       510       520       530
a   DRMKREFNVEANVGKPQVAYRETIRQKVTDVEGKHAKQSGGRGQYGHVVIDMYPLEPGSN
i   DRMRREFKVEANVGKPQVAYRETIRNTVKDIEGKHAKQSGGRGQYGHVVIDMYPLEEG..
c   DRMKREFSVDCNVGKPQVAYRETIRG.KAEVEGKFVRQSGGRGQYGHVWVKLEPSEPG..
h   DRMKREFSVDCNVGKPQVAYRETIRG.KAEVEGKFVRQSGGRGQYGHVWIKLEPSEPG..
d   DRMRREFNVEANIGKPQVSYRERITKN.CEIEGKFVRQSGGRGQFGHCWIRFAPADEGQ.
g   DRMRREFNVEANIGKPQVSYRERITKN.CEIEGKFVRQSGGRGQFGHCWIRFAPADEGQ.
f   DRMKREFNVECNVGKPQVSYREKITKSNVEIEGKFVRQSGGRGQFGHCWIRFSEPDVDEK
j   DRMKREFGVEANIGKPQVAYRETITKDNVEIEGKFVRQSGGRGQFGHCWIRFSAADVDEK
b   DRMKKEFNVECNVGAPMVSYRETFKSS.AQVQGKFSRQSGGRGQYGDVHIEFTPNETGA.
e   DRMRREFRVEANVGDPQVSYRETFRKS.AQVEGKFVRQSGGRGQYGHVWIEFGPNEEGK.
```

```
                   540       550       560       570       580
a   ...PKGYEFINDIKGGVIPGEYIPAVDKGIQEQLKAGPLAGYPVVDMGIRLHFGSYHDVD
i   ....KAYEFVNDIKGGVIPGEFIPGVDKGIREQLKSGPLAGYPVMDLGVRLHFGSYHDVD
c   ....EGFVFVDEIVGGVIPKEYISSVSKGIEEQMNSGVLAGYPVLDIKATLFDGSYHDVD
h   ....EGFVFVDEIVGGVVPKEYISSVSKGIEEQMNSGVLAGYPVLDIKATLFDGSYHDVD
d   ....EGLQFVNEVVGGVVPKEYIPAIQKGIEEQMKNGVVAGYPLIGLKATVFDGSYHDVD
g   ....EGLQFVNEVVGGVVPKEYIPAIQKGIEEQMKNGVVAGYPLIGLKATVFDGSYHDVD
f   GNITEGLVFTNEVVGGVIPKEFIAPIQKGIEEQMKNGVVAGYPLLGLKATVFDGSYHDVD
j   GNITEGLVFENEVVGGVVPKEYIPAIQKGIEEQMKNGVVAGYPLIGLKATVFDGSYHDVD
b   .....GFEFENAIVGGVVPREYIPSVEAGLKDAMENGVLAGYPLIDVKAKLYDGSYHDVD
e   .....GFEFENAIVGGVVPREYIPAVQAGLEGALDNGVLAGYPLIDIKAKLYDGSYHDVD
```

```
    590       600       610       620       630       640
a   SSELAFKLAASIAFKEGFKKAKPVLLEPIMKVEVETPEENTGDVIGDLSRRRGMLKGQES
i   SSELAFKIAASMAFKAGFMKANPVLLEPIMKVEVETPEDYMGDVIGDLNRRRGLIEGMED
c   SSEMAFKIAGSMAFKKGALEAQPVILEPMMNVEVTTPEDWMGDVVGDLNRRRGIIEGMDE
h   SSEMAFKIAGSMAFKKGALEAQPVILEPMMNVEVTTPEDWMGDVVGDLNRRRGMIEGMDE
d   SNEMAFKVAASMATKQLAQKGGGELLEPIMAVEVVTPEDYMGDVMGDLNRRRGMILGMED
g   SNEMAFKVAASMATKQLAQKGGGELLEPIMAVEVVTPEDYMGDVMGDLNRRRGMILGMED
f   SNEMAFKIAASMATKQLAQKGGGVVLEPIMKVEVVTPEDYLGDVMGDLSRRRGMIQGNED
j   SNEMAFKIAASMATKQLAQKGGGKVLEPIMKVEVVTPEDYMGDVMGDLNRRRGLIQGMED
b   SSEMAFKIAASLALKEAAKKCDPVILEPMMKVTIEMPEEYMGDIMGDVTSRRGRVDGMEP
e   SNEMAFKVAASMALRNAAKKCDPVILEPMMAVEVVIPEEYLGDIMGNITSRRGRVDGMEA
```

```
    650       660       670       680       690       700
a   EVT.GVKIHAEVPLSEMFGYATQLRSLTKGRASYTMEFLKYDEAPSNVAQAVIEARGK..
i   GPS.GKIVRALVPLAEMFGYATALRSATQGRASYAMEFAKYHDAPTNVAQAVIEERKSK.
c   GVAGLKIIRAQVPLSEMFGYATDLRSATQGRASYSMEFHEYAEVPKNFADKIIAERGY..
h   GVAGLKIIRAQVPLSEMFGYATDLRSATQGRASYSMEFNEYAEVPKNFADKIIADRGY..
d   TVS.GKVIRAEVPLGEMFGYATDVRSMSQGRASYSMEFKKYNTAPAHIAETVSKKQG...
g   TVS.GKVIRAEVPLGEMFGYATDVRSMSQGRASYSMEFKKYNTAPSHIVETVTKKQG...
f   SVS.GKVITAEVPLGEMFGYATDVRSMSQGRASYSMEFSKYAEAPSNIVEALVKKQG...
j   TVS.GKVIRAEVPLGEMFGYATDVRSMSQGRASYSMEFSKYAEAPSNIVEALVKKQG...
b   RGN.AQVVNAYVPLSEMFGYATSLRSNTQGRGTYTMYFDHYAEVPKSIAEDIIKKNKGE.
e   RGN.AQVVRAFVPLANMFGYATHLRSGTQGRGVYTMQFDHYEEVPKSIAEEIIKANGGNN
```

**a**	. . .
**i**	. . .
**c**	. . .
**h**	. . .
**d**	. . .
**g**	. . .
**f**	. . .
**j**	. . .
**b**	. . .
**e**	KED
